# A data-driven approach for optimal design of integrated air quality monitoring network in a chemical cluster

**DOI:** 10.1098/rsos.180889

**Published:** 2018-09-05

**Authors:** Zhengqiu Zhu, Bin Chen, Sihang Qiu, Rongxiao Wang, Yiping Wang, Liang Ma, Xiaogang Qiu

**Affiliations:** 1 College of System Engineering, National University of Defense Technology, 109 Deya Road, Changsha 410073, People's Republic of China; 2 Faculty of Electrical Engineering, Web Information Systems, Mathematics and Computer Science, TU DELFT 2628 XE Delft, The Netherlands; 3 The Naval 902 Factory, Shanghai, People's Republic of China

**Keywords:** Bayesian maximum entropy, multi-objective optimization model, air quality monitoring network, atmospheric dispersion simulation system

## Abstract

The chemical industry is of paramount importance to the world economy and this industrial sector represents a substantial income source for developing countries. However, the chemical plants producing inside an industrial district pose a great threat to the surrounding atmospheric environment and human health. Therefore, designing an appropriate and available air quality monitoring network (AQMN) is essential for assessing the effectiveness of deployed pollution-controlling strategies and facilities. As monitoring facilities located at inappropriate sites would affect data validity, a two-stage data-driven approach constituted of a spatio-temporal technique (i.e. Bayesian maximum entropy) and a multi-objective optimization model (i.e. maximum concentration detection capability and maximum dosage detection capability) is proposed in this paper. The approach aims at optimizing the design of an AQMN formed by gas sensor modules. Owing to the lack of long-term measurement data, our developed atmospheric dispersion simulation system was employed to generate simulated data for the above method. Finally, an illustrative case study was implemented to illustrate the feasibility of the proposed approach, and results imply that this work is able to design an appropriate AQMN with acceptable accuracy and efficiency.

## 1. Introduction

Long-term violations of air quality standards with respect to industrial production emissions have posed a great threat to the management of a chemical industrial cluster. Indeed, the by-products produced during the chemical production processes are noxious, even sometimes highly toxic, and they are often discharged to the nearby atmospheric environments without purification treatment. As a result, the air quality in developing countries, where the control on industrial pollution is absent or very low, is extremely poor [1], leading to substantial health problems for the residents [2] and to the potential destruction of the ecosystem. Therefore, it is urgent for environmental authorities to come up with effective atmospheric pollution-controlling measures that can ensure safe production, provide intelligent decisions and maintain social stability [3].

Faced with this situation, governments in developing countries have introduced a series of measures to abate atmospheric pollution [4,5]. These measures include promulgation of standards, norms and emergency plans for environmental quality as well as air quality monitoring and control [6]. One of the most substantial tasks is the creation of an air quality monitoring network (AQMN) for detecting and monitoring the disposed atmospheric pollutants in the chemical cluster by environmental protection authorities. Objectives and necessities of such monitoring networks are reported frequently in the literature [7–11] and can be summarized as follows: (i) objectives related to air pollution legislation, long-term plan of land use and the announcement of emergency situations; (ii) the evaluation of exposure of the population and other potential receptors; (iii) the controlling of emissions from significantly important sources (e.g. thermal power plant); and (iv) analysis of air pollution data to determine emission trends in air pollution or for further research.

Moreover, the minimization of network cost to accomplish these objectives has been always reinterpreted as a constraint on the available budget [6,12]. Previous methods in the literature lacked in accomplishing the task of designing a network capable of fulfilling all of the objectives above. Most of the reported methods applied to specific situations wherein one or two of the previous objectives are considered [13].

Generally, existing methods of establishing an AQMN typically consider parameters related to ambient concentrations of gaseous pollutants, such as atmospheric transport and dispersion, diffusion source characteristics, secondary reactions, deposition characteristics and local topography [14]. The objective of these network design methods is usually aimed at identifying the sites of maximum contaminant concentration, maximum contaminant dosage and maximum population protection as well as covering the maximum urban areas with the minimum number of monitoring stations [7,15–20].

Among the previous works on an AQMN design, Goldstein [21] designed an AQMN in the Greater London area based on the concept of a spatial correlation analysis. A statistical measure of information content was used to evaluate the availability of a particular AQMN in Canada [22]. Moreover, interpolation techniques were taken in The Netherlands when assessing the errors in the spatial analysis [23]. Afterwards, air quality simulation models and population exposure information were applied to generate representative combined patterns; and then McElroy *et al*. applied the concepts of ‘sphere of influence' (SOI) and ‘figure of merit' (FOM) to determine the minimum numbers of monitoring stations required in urban areas [24]. A methodology which involves the multiple-criteria method, a spatial correlation technique in conjunction with a fuzzy analytic hierarchy process, was also used to determine the optimum number of ambient air quality stations [12]. To sidestep the problem of specifying a particular design objective or some conflicted objectives, Le & Zidek [25] proposed an entropy-based Bayesian optimization method to maximize the uncertainty reduction when selecting a number of stations. Later on, Ainslie *et al*. applied the method of Le and Zidek to the spatial redesign of the AQMN with a two-decade-long dataset [26]. It could be drawn that most of these methods focused on optimizing the number and layout of fixed monitoring stations for urban areas and most of these methods required complete sequence datasets.

However, the ambient air quality monitoring problem in our work is totally different from the domains of research mentioned above. In this paper, a data-driven method is introduced to design an integrated AQMN wherein both high-accuracy air quality monitoring stations and gas sensor modules are modelled for a chemical cluster. Owing to the lack of long-term measurements, our developed atmospheric dispersion simulation system is used to generate a simulated historical dataset based on the results of trends analysis. The dataset collected by gas sensor modules cannot guarantee completeness and continuity when considering the actual situation. Thus, Bayesian maximum entropy (BME) is introduced to generate the predicted concentration distribution of gaseous pollutants under this condition of the dataset. Finally, a multi-objective optimization model (i.e. maximum concentration detection capability (CDC) and maximum dosage detection capability (DDC)) aimed at optimizing the monitoring network of gas sensor modules is built up on account of the predicted concentration distribution over the study area. When latest measurements of gas sensor modules are imported, the monitoring network would be redesigned accordingly after conducting the BME process and multi-objective optimization process. In view of that, the number and the initial distribution of gas sensor modules have influenced the prediction accuracy greatly [27–29], thus they are also discussed in the results section. The proposed data-driven method, which involves two kinds of inspection resources, is a supplement of existing monitoring approaches and greatly improves the validity of monitoring data.

The remainder of this paper is organized as follows: §2 presents the main modelling process of the proposed data-driven method. Afterwards, datasets and experimental set-ups are elaborated in §3. Subsequently, results and discussions are conducted in §4. Finally, conclusions and future lines of research are discussed in §5.

## 2. Methods

In this section, a data-driven approach based on the BME method is presented to acquire the spatio-temporal concentration distribution of gaseous pollutants in a chemical cluster by importing long-term monitoring data from multiple monitoring sites. With the spatiotemporal distribution of airborne contaminants, the optimal layout of gas sensor modules can be determined based on different optimization targets. Together with the fixed monitoring stations, an integrated monitoring network is built up to conduct valid monitoring of gaseous pollutants in a chemical cluster.

Long-term real measurements of gas sensor modules are absent in this study, and thus it is impossible to conduct BME analysis with real data. Fortunately, monitoring measurements collected by fixed monitoring stations in conjunction with historical meteorological data can be applied to estimate the parameters of the diffusion source term (i.e. location and release rate) of a particular gaseous pollutant through source estimation methods. Interested readers are referred to our previous research works [30–32], wherein more technical and theoretical details are introduced. Then, the source term as well as historical meteorological data is imported to our developed atmospheric dispersion simulation system as inputs. After simulation, the generated concentration distribution of a particular pollutant serves as the simulated historical concentration distribution data. Moreover, the concentration data extracted at locations of gas sensor modules are taken as the historical monitoring measurements and would serve as inputs in BME analysis.

### 2.1. KD atmospheric dispersion simulation software

To simulate the atmospheric dispersion process of gaseous pollutants, the KD atmospheric dispersion simulation system (KD-ADSS) is used to model airborne particle diffusion in the atmosphere. Specifically, KD-ADSS is a Gaussian model-based simulation system developed by National University of Defense Technology. The simulation tool has been validated by the commercial software PHAST, the Indianapolis field study and a study of the Fukushima Dai-ichi nuclear accident. Moreover, the system uses a series of puffs to approximate an airborne plume of gaseous pollutants. It is essential to denote by $g(t,\;z,\;\theta ,\;t_{s})$ the dispersion function of a single puff with a unit release rate, where *t_s_* means the time when the puff starts to disperse; *θ* = {*l*, *q*(*t*)} represents the release source formed by source location *l* and release rate *q*(*t*); and t, z denote, respectively, the time and location used to calculate the theoretical concentration.

KD-ADSS also supports wind field generation and meteorological data import via the inverse distance weighted (IDW) method [33]. The temporal interval between each puff is *δ*, so the expression of function f (t, z, θ) can be written as follows:

2.1 $$f\;(t,\;z,\;\theta )\approx \sum_{i=0}^{n-1}q_{i}g(t,\;z,\;\theta ,\;i\delta ),$$

where *q_i_* denotes the mass of airborne contaminant of puff *i*; and *n* is the number of puffs, which satisfies that the release duration equals *n* × *δ*. Moreover, the following relation should be achieved.

2.2 $$\int_{i\delta }^{(i+1)\delta }q(t)\;\text{d}t=q_{i}.$$

### 2.2. Bayesian maximum entropy

In air quality studies, the concentration distribution of a particular pollutant, such as sulfur dioxide (hereafter, this particular pollutant serves as the research subject), is represented in the form of a spatio-temporal random field (**S/TRF**) *X*(***p***), which assumes values at space/time points p=(s, t), where ***s*** is the location vector, while *t* denotes time [34]. Generally, environmental protection authorities are more concerned with the estimation values of a particular pollutant at unmeasured locations through analysing available datasets and physical knowledge. The estimation process leads to a spatio-temporal map which presents the concentration distribution of a specific pollutant in space and time. BME, a space–time data analysis method in a modern statistical framework introduced by Christakos [34,35], provides an effective, efficient and accurate way for the estimation of concentration distribution of gaseous pollutants. It is worth noting that BME has already been proved to perform much better in a spatio-temporal analysis compared to the Kriging technique and interpolation approaches, especially in a situation where the dataset cannot guarantee completeness and continuity [36].

**Figure 1.**
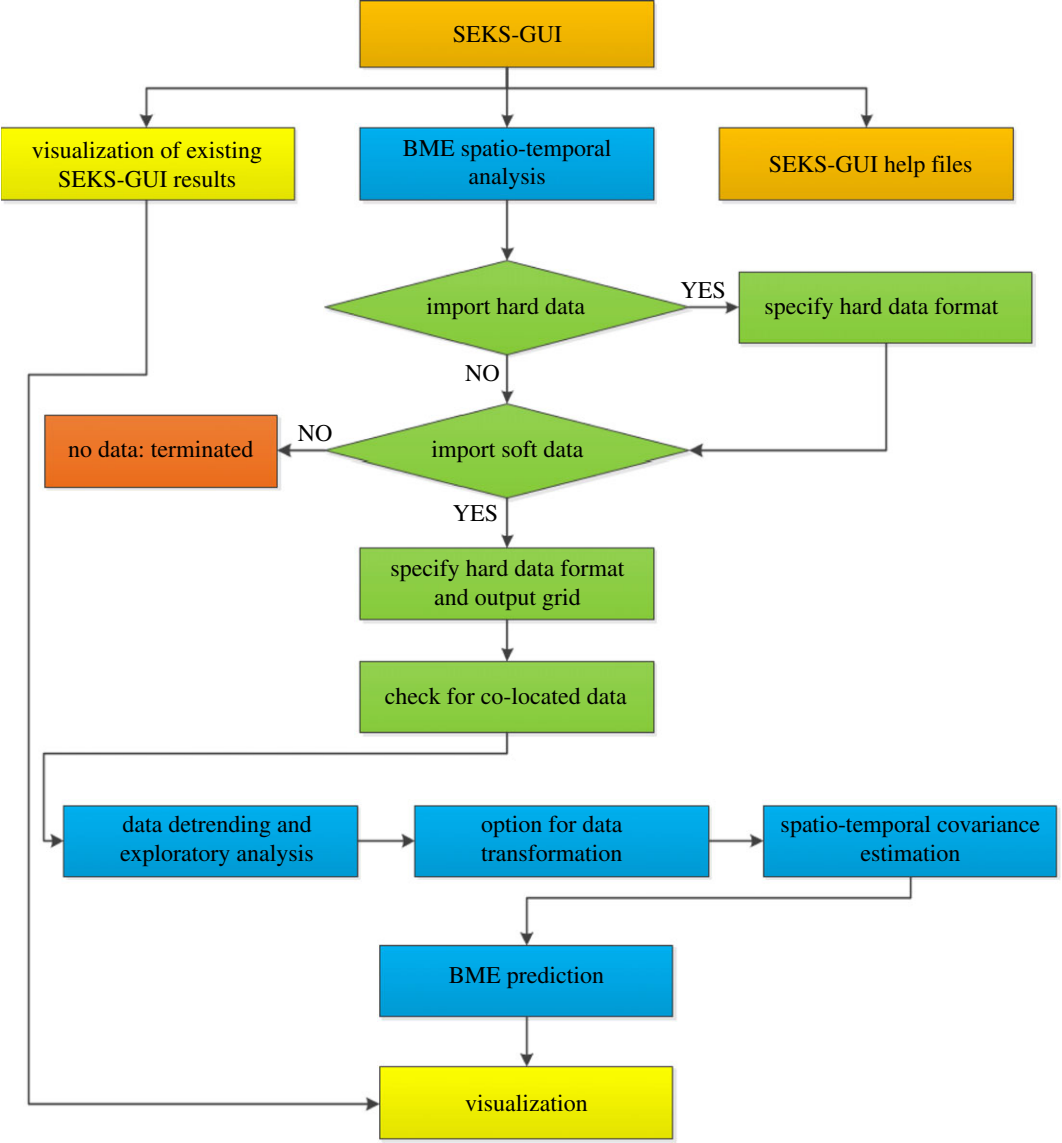
Workflow of BME analysis in SEKS-GUI.

The publicly available SEKS-GUI software library [37] (i.e. a non-commercial Matlab-based software package) is used in this paper to implement the space–time BME analysis. The software solves the fundamental BME equations of spatio-temporal dependence analysis and mapping as follows [38] and the following figure 1 shows the flowchart of how the BME analysis works in SEKS-GUI.

2.3 $$\begin{array}{c} d\chi (g-\bar{g})e^{\mu^{T}g}=0\\ d\chi \xi_{s}e^{\mu^{T}g}-Af_{K}(p)=0, \end{array}\}$$

where ***g*** is a vector of the *g_a_*-function (*α* = 1, 2,…) and $\bar{g}$ denotes the statistical expectation; *μ* is a vector of *μ_a_*-coefficients that depends on the space–time coordinates and is related to ***g*** (i.e. the *μ_a_* indicates the relative significance of each *g_a_*-function in the composite solution sought); the *ξ_s_* represents the site-specific knowledge bases available; *A* is a normalization parameter; and *f_K_* is the attribute probability density function (PDF) at each point. The parameters of ***g*** and *ξ_s_* are inputs to the equation, whereas the unknowns are the parameters of *μ* and *f_K_* across space and time.

BME analysis has a wide range of applications because the fundamental equations make no restrictive assumptions about the underlying probability distributions (i.e. non-Gaussian laws are automatically incorporated) and the shape of the space–time predictor (i.e. nonlinear predictors are allowed). Therefore, the BME framework is able to handle with a broader scope of knowledge bases (KB) types and uncertain data [39].

General KB (*G*-KB) and site-specific KB (*S*-KB) are integrated in the fundamental BME equations. Generally, the *G*-KB includes physical laws, theoretical models of space–time dependence (e.g. covariance, semivariogram etc.), empirical relations and logic-based assertions that are concerned with the pollution *X*(***p***). The *S*-KB usually consists of observed hard data and soft data (i.e. measurements with a significant amount of uncertainty). In our study, the *G*-KB contains theoretical covariance models, while the *S*-KB includes airborne pollutant data.

SEKS-GUI represents the prediction grid by the space–time vectors ***p****_k_*, in which case the fundamental BME equations compute the complete prediction PDF *f_K_* at each ***p****_k_*. After determining the objective of study and PDF *f_K_*, predictions of *X*(***p***) can be derived at each spatio-temporal node ***p****_k_* of the mapping grid.

The software thus generates informative airborne pollutant maps that completely cover the spatial and temporal continua within their respective extents and also enable the establishment of an optimization model in the next subsection.

### 2.3. Integrated air quality monitoring network

In this subsection, two objectives are proposed to optimize the design of an AQMN of gas sensor modules based on the results of BME analysis. In conjunction with the fixed monitoring stations, an integrated AQMN with the ability of global and effective inspection in a chemical cluster is built up.

#### 2.3.1. Maximum concentration detection capability

Maximum concentration detection capability (CDC) is defined as the most frequent polluted grids captured by an optimal AQMN of gas sensor modules wherein the pollutant levels are remarkably exceeding the threshold of the standard value or mean value. The model, based on this objective, is established as follows:

2.4 $$\mathrm{Max}\;o_{\mathrm{CDC}}=\sum_{i=1}^{T}d_{i},$$

2.5 $$\;\text{such that}\;\;d_{i}=\sum_{\;j\in M_{i}}y_{j}\;\forall i,$$

2.6 $$\sum_{\;j=1}^{J}y_{j}\leq Q$$

2.7 $$\;\text{and}\;y_{j}\in \{0,\;1\}\;\forall j,$$

where the notation of *d_i_* is a variable, indicating the number of grids where the pollutant level exceeding the threshold of the standard value is detected in the *i*th month; *T* is the total number of months in a year; *y_j_* is a binary integer that indicates whether a gas sensor is placed in grid *j*; *J* denotes the total number of grids in the study area; *M_i_* is the set of grids in the *i*th month with a pollutant level greater than the threshold of the standard value or mean value; and *Q* is the upper limit of the number of gas sensor modules in an AQMN.

#### 2.3.2. Maximum dosage detection capability

Some areas may have a low incidence of high-level pollution but a large dosage due to long-term exposure [40]. Therefore, using the previously mentioned objective of CDC alone when designing an AQMN may be inadequate. The objective and the model for maximum DDC can be formulated as follows:

2.8 $$\mathrm{Max}\;o_{\mathrm{DDC}}=\sum_{\;j\in N}y_{j},$$

2.9 $$\;\text{such that}\;C_{j}=\sum_{i=1}^{T}C_{ij},$$

2.10 $$j\in N,\;\text{if}\;C_{j}\geq \frac{\sum_{\;j=1}^{J}C_{j}}{\left|J\right|},$$

2.11 $$\sum_{\;j=1}^{J}y_{j}\leq Q$$

2.12 $$\;\text{and}\;y_{j}\in \{0,\;1\}\;\forall j,$$

where the notation of *C_ij_* represents the pollutant level at grid *j* in the *i*th month. The first and second constraints indicate that the notation of *N* is the set of grids with an accumulated dosage pollutant level greater than the threshold of the mean value. The third and fourth constraints are the same as those defined in the upper subsection.

Of these two single objective models described above, the CDC and DDC can be applied independently or combined into a multi-objective model. In this paper, the combination of the two objectives seems to have little impact on the final result in our experiments. Therefore, we use the different single objective models to design an AQMN of gas sensor modules, respectively.

## 3. Datasets and experimental set-ups

In this section, datasets and experimental set-ups used in this research are elaborated in detail, consisting of the real datasets collected from the Shanghai chemical cluster, simulated datasets generated by KD-ADSS, set-up of the study area and set-up of the SEKS-GUI software library.

### 3.1. Actual datasets

The actual dataset was collected by the environmental protection authority in the Shanghai chemical cluster, including up to 109 categories of airborne pollutants as well as meteorological data from December 2015 to November 2016 at five fixed monitoring stations. These air quality monitoring stations were constructed according to the regional project for air quality conservation, established by empirical judgement and the governmental law [4]. After projecting the WGS84 geographical coordinates into UTM Cartesian coordinates, the resulting locations of these monitoring stations are listed in table 1 with some additional information.

**Table 1.** Cartesian coordinates of fixed monitoring stations and additional information. (no. denotes the English number of monitoring stations used in the following figure; *X*, *Y* represents the UTM Cartesian coordinates of monitoring stations; explanation denotes the English name of these monitoring stations; and code represents the serial number loaded in our database).

no.	*X*	*Y*	explanation	code
A	−1022.9	223.0	northwest station	S02101
B	−796.9	−431.5	Secco station	S02104
C	3575.3	3518.9	northeast station	S02102
D	1620.6	1293.3	union road station	S02103
E	4151.1	1292.3	Covestro station	S02015

High-accuracy reactors contained in these fixed monitoring stations are able to collect up to 118 categories of airborne pollutants (e.g. VOCs, NO*x*, SO_2_ etc.). The measurement interval of these facilities lasts no longer than a few seconds, which can be considered as continuous collections. Therefore, these data are suitable for trend analysis (e.g. hourly average, daily average, monthly average and annual average analysis), which can provide substantial information for the following experiment set-ups. The total amount of the dataset is 20 430 248 entries, while the available data contain 20 290 164 entries. The ineffective data were associated with the sites where temporary failures, warm-up of devices or stations working on a non-systematic temporal basis existed.

### 3.2. Simulated datasets

To simulate dispersion scenarios of a particular gaseous pollutant (e.g. sulfur dioxide), the information of the emission source *θ* (i.e. the location of the emission source and the release rate of the emission source)**,** meteorological parameters (i.e. wind direction *d* and wind speed *v*) and the environmental parameters (e.g. atmospheric stability and terrain type) must be considered.

**Figure 2.**
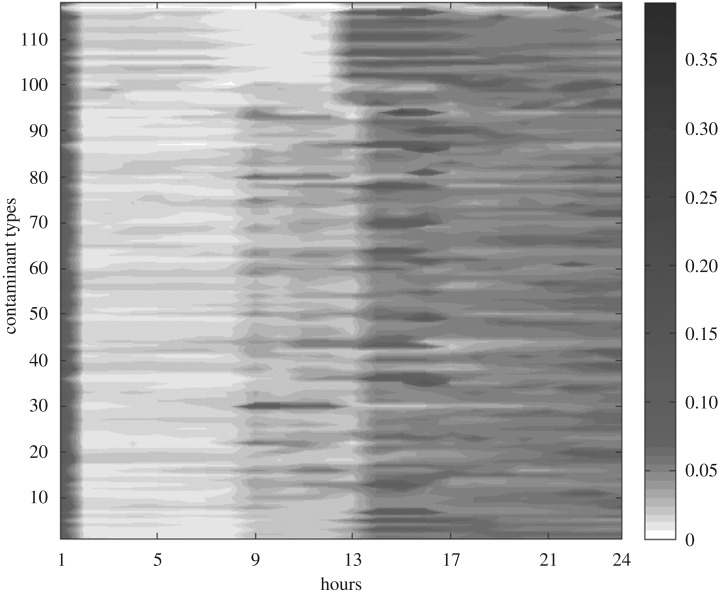
Hourly averaged concentration trend during the past year in the Shanghai chemical cluster. (The concentration is measured in μg m^−3^; The *X*-axis is the time series of 1 day, while the *Y*-axis represents the main atmospheric contaminants monitored by monitoring stations. The background colour of this figure is white, which means the concentration value of atmospheric pollutants is zero; moreover, a darker area represents higher gas concentration.)

**Figure 3.**
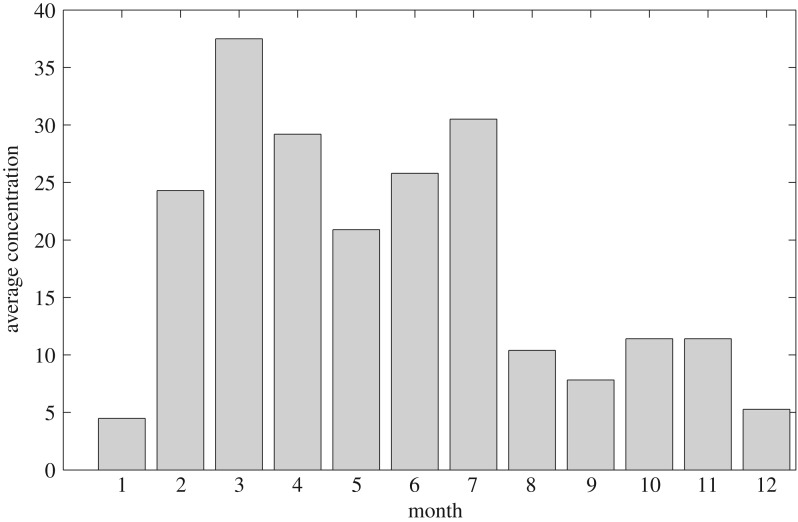
Monthly averaged concentration trend of a particular air pollutant SO_2_. (The concentration is measured in μg m^−3^; the *X*-axis is the time series of a year, while the *Y*-axis represents the averaged concentration of a particular air contaminant SO_2_ monitored by monitoring stations.)

Our previous works on source estimation methods [30–32] enable us to predict the locations of emission sources and the release rates of emission sources at the same time. However, the prediction accuracy of the release rate is rather low compared with that of location. Therefore, the release rates of the predicted emission spots are assumed to vary from 0 to 5 g s^−1^ according to the results of hourly averaged concentration trend analysis and monthly averaged concentration trend analysis as shown in figures 2 and 3 based on actual datasets. It can be concluded from figure 2 that the discharging by chemical plants clearly has temporal characteristics. The discharging amount of atmospheric pollutants in the time unit of 12–24 h is far greater than that in the time unit of 0–12 h. Thus, the release rate during the period of 12–24 h is set twice of that during the period of 0–12 h in simulation. Moreover, through analysing the monthly averaged concentration data of SO_2_, it is concluded from figure 3 that the discharging amount of SO_2_ from February to July is greater than that during the period from August to January. Therefore, the value of the release rate in summer months is set larger than that in winter months.

Gaussian diffusion coefficients *σ_y_*, *σ_z_*, posing a substantial impact on dispersion of a particular air contaminant, can be expressed as follows [41]:

3.1 $$\begin{array}{c} \sigma_{y}(D_{x})=a_{y}D_{x}{\left(1+b_{y}D_{x}\right)}^{-c_{y}}\\ \sigma_{z}(D_{x})=a_{z}D_{x}{\left(1+b_{z}D_{x}\right)}^{-c_{z}}, \end{array}\}$$

where *D_x_* denotes the downwind distance of the interest point; the parameters of *a_y_*, *b_y_*, *c_y_*, *a_z_*, *b_z_*, *c_z_* depend on the environmental conditions (i.e. atmospheric stability and terrain type, as shown in table 2). According to the terrain type (i.e. urban) and atmospheric stability (i.e. D) of the Shanghai chemical cluster, the actual values of these parameters in the third row are selected.

**Table 2.** Relationships between diffusion coefficients and atmospheric stability. (The terrain type of the study area in this research is urban and the most frequent atmospheric stability is assumed to be D).

terrain type	class of atmospheric stability	*a_y_*	*b_y_*	*c_y_*	*a_z_*	*b_z_*	*c_z_*
urban	A and B	0.32	0.0004	−0.5	0.24	0.001	−0.5
urban	C	0.22	0.0004	−0.5	0.2	0	−0.5
urban	D	0.16	0.0004	−0.5	0.14	0.0003	−0.5
urban	E and F	0.11	0.0004	−0.5	0.08	0.0015	−0.5
open country	A	0.22	0.0001	−0.5	0.2	0	−0.5
open country	B	0.16	0.0001	−0.5	0.12	0	−0.5
open country	C	0.11	0.0001	−0.5	0.08	0.0002	−0.5
open country	D	0.08	0.0001	−0.5	0.06	0.0015	−0.5
open country	E	0.06	0.0001	−0.5	0.03	0.0003	−1
open country	F	0.04	0.0001	−0.5	0.16	0.0003	−1

Finally, all the input data including the emission sources, historical meteorological data, environmental parameters and Gaussian diffusion coefficients are imported to conduct hourly dispersions for a decade in KD-ADSS. Thus, a total of approximately 86 400 dispersion scenarios (i.e. 24(h) × 30(day) × 12(month) × 10(year)) are obtained. Further, the simulated historical dataset is used to produce monthly averaged SO_2_ measurements at a total of 60 monitoring locations of gas sensor modules. Finally, the first 9-year monthly averaged measurements at monitoring locations of gas sensor modules as well as geospatial data would be imported to conduct the BME analysis and generate the prediction measurements at unmeasured locations in the 10th year.

### 3.3. Experimental set-ups

#### 3.3.1. Set-up of study area

**Figure 4.**
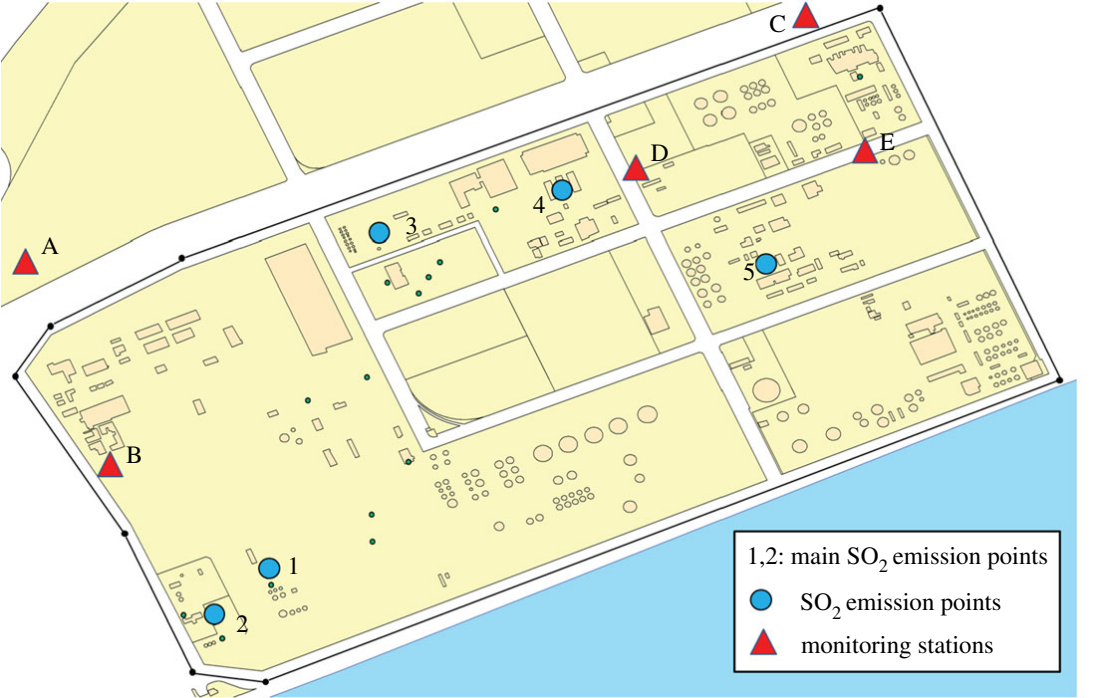
Concise map of the research area.

**Figure 5.**
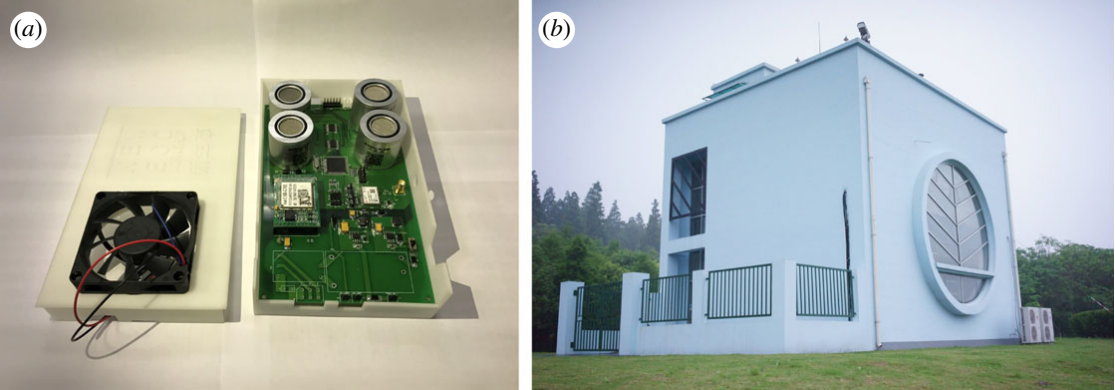
Inspection resources of inspection agency. (*a*) The gas sensor modules and (*b*) one of the high-accuracy air quality monitoring stations.

Similar to previous research [31,42], a chemical cluster in Shanghai is selected as our study area in this paper. Figure 4 shows a concise GIS map of a chemical cluster in Shanghai, China. Through investigating the emissions of SO_2_ and referring to the main by-products information of chemical plants, five possible SO_2_ emission sources are located in this area. On the map, the 19 small circles are the complete set of discharge points for all contaminants, among which the five blue circles are the SO_2_ discharge spots. After projecting the WGS84 geographical coordinates into UTM Cartesian coordinates, the resulting locations of all the candidate sources are listed in table 3 with concrete information. Moreover, the triangles indicate the fixed high-accuracy air quality monitoring stations; and the area marked by the black quadrilateral box is the main working area of this district, while the area beneath the working area is the sea. Meanwhile, an illustrative picture of the two inspection resources is shown in figure 5. The gas sensor modules [43] (the sensor probes are produced by Alphasense—The Sensor Technology Company) used in this research are provided by SINGOAN Electronic Technology Co., Ltd, while the high-accuracy monitoring stations [44] are the product of Beijing Safety Equipment Manufacturing Co., Ltd. These inspection resources (i.e. five monitoring stations and a total number of 60 gas sensor modules) are operating to inspect 55 chemical plants. It is worth noting that the high-accuracy monitoring stations as well as gas sensor modules are regularly calibrated by technical staff or automatic calibration devices to ensure the valid collection of monitoring data. In addition, it is also worth noting that the generated measurements of gas sensor modules would be considered as hard data in this paper when used in the BME analysis. However, in real circumstances, a certain proportion of the dataset collected by gas sensor modules should be considered as soft data because of the incompleteness and discontinuity in data. The monitoring data of the five fixed monitoring stations are used as inputs in source estimation methods when determining the main emission sources of SO_2_. Through calculation and our investigation, the chimney of the sulfuric acid recovery (SAR) system and the waste incinerator for acrylonitrile (AN) were the major locations contributing to the emission of SO_2_. Then, the source term and historical meteorological data are imported into KD-ADSS to generate simulated historical concentration data of SO_2_ within the area of the Shanghai chemical cluster in §3.2.

**Table 3.** Cartesian coordinates of SO_2_ emission points and additional information. (no. denotes the serial number of emission spots; *X*, *Y* represents the UTM Cartesian coordinates of emission spots; explanation denotes the English name of these emission spots; and main contaminants represents the by-products generated by different emission spots).

no.	*X*	*Y*	height	explanation	main contaminants
1	−132.575	−1317.63	50	waste incinerator for acrylonitrile (AN)	SO_2_, NO*x*, VOCs, NH_3_
2	−302.901	−1483.42	68	chimney of sulfuric acid recovery (SAR) system	SO_2_, NO*x*, vitriol fog
3	267.1415	0.359916	27	furnace no. 1	PM_2.5_, PM_10_, SO_2_
4	861.3643	147.0462	27	furnace no. 2	PM_2.5_, PM_10_, SO_2_
5	1532.017	−142.542	30	hazardous waste incinerator	CO, SO_2_, NO*x*, PM_2.5_, PM_10_, HF, HCl, dioxin

**Figure 6.**
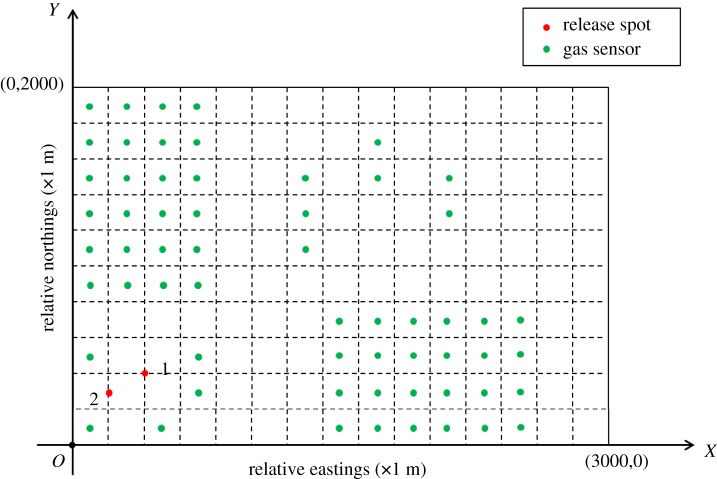
Simplified study area used in experiments. (The grid size in this figure is 200 metres; red points denote main release spots of SO_2_; green points represent the initial layout of gas sensor modules based on wind field analysis).

**Figure 7.**
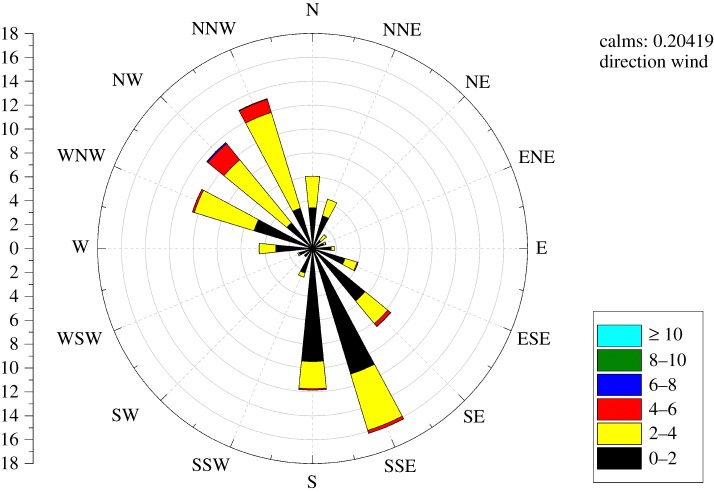
Annual wind-rose diagram for the research area. (The left vertical axis indicates the proportion of a specific direction of wind versus all directions of wind, while the right axis in the bottom of this figure illustrates that a specific colour in a wind direction denotes the corresponding interval of wind speed. Moreover, it is noteworthy that the prevailing wind in the Shanghai chemical cluster is in the northwest and southeast directions. Generally, most concentration distribution of a specific gaseous pollutant is distributed in the downward direction of the wind. Therefore, it is possible for researchers to predict the approximate distribution of the gaseous pollutants in a chemical cluster through analysing the regular variation of wind speed and wind direction. Therefore, most of the gas sensor modules are initially positioned in the northwest and southeast part of the Shanghai chemical cluster in this study.)

The study area of the Shanghai chemical cluster can be further simplified as a 2000 × 3000 m rectangle on account of experimental conditions. The illustration of this simplified study area is shown in figure 6. Through importing monitoring measurements of the five fixed monitoring stations as well as historical meteorological data into the source estimation method, main release spots in the past year were calculated. Specifically, the red circles represent the two most frequent release spots calculated by the source estimation method (1 denotes the waste incinerator for AN and 2 denotes the chimney of the SAR system). The approximate locations of these two release spots are at (400 m, 400 m) and (200 m, 300 m), respectively. Moreover, the rectangle of the study area is divided into 150 quadrate grids wherein the grid size is 200 × 200 m. The initial layout of the gas sensor modules is designed as shown in figure 6 according to the results of wind field analysis (the analysis result is exhibited in figure 7). In addition, it is assumed that all of the gas sensor modules are positioned at the height of 20 m in the centre of each grid.

#### 3.3.2. Set-up of SEKS-GUI software library

SEKS-GUI implements the BME methodology for spatio-temporal analysis. The workflow of how this analysis works in SEKS-GUI is presented as follows.

Firstly, the hard data information with exact measurements (i.e. the generated monitoring measurements at monitoring locations from the first 9-year simulated dataset), the soft data with certain uncertainty (i.e. the soft data are absent in this study) and output GIS grid (i.e. information about the study area) are imported into SEKS-GUI. Then, the data are detrended and brought from raw input information into suitable processing form. For the detrending process, Gaussian kernel smoothing is applied across the dataset. Moreover, a data transformation aimed at reshaping the detrended dataset from the original space of values (original-space) into a space where their distribution resembles a Gaussian one (transformation-space) is implemented. Subsequently, a covariance analysis is conducted to investigate correlation patterns among the data in the next stage. Finally, we have to select and initialize the type of BME prediction. Four different prediction types are offered in the software library, namely, BME mode, BME moments, BME PDF as well as BME confidence intervals and they are ranked with respect to the time and complexity required for the computations, starting with the fastest and simplest one and ending with the most time-consuming and complicated. In this study, the most time-consuming and complicated type (i.e. BME confidence intervals) is chosen to improve the prediction accuracy. Most importantly, confidence interval option is of vital importance to the predicted results. Setting a higher confidence will bring users more accurate predicted values, but may also lead to the failure of prediction on some grids because the predicted results cannot pass the confidence testing. By contrast, setting a lower confidence will ease the computation burden and provide predicted results at all grids, but may also lead to unacceptable predicted values at low accuracy level. In this paper, 95 percentage of confidence level is selected. In visualization, SEKS-GUI offers a bundle of mapping options to display the BME prediction results once the BME output MAT-file is loaded. In this study, the mean of the prediction posterior PDF at each output grid node is the main focus.

Moreover, apart from the visualization results, SEKS-GUI also offers the option to download the available data during the calculation process for further research. In this study, the available data are used to design the optimal AQMNs.

## 4. Results and discussion

### 4.1. Results of atmospheric contaminants dispersion simulation

**Figure 8.**
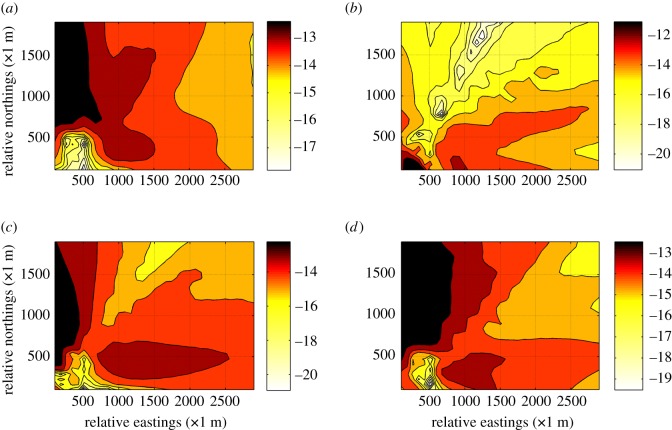
Monthly mean concentration distribution of SO_2_ in 2016 generated by KD-ADSS. (*a*) Distribution in January; (*b*) distribution in June; (*c*) distribution in July; (*d*) distribution in December.

To acquire the simulated mean concentration distribution of SO_2_ during the past 10 years, about 86 400 dispersion scenarios based on historical meteorological data were run through our developed KD-ADSS. Figure 8 shows the simulated mean concentration distribution of SO_2_ in January, June, July and December of 2016. To better exhibit the dispersion effect, the concentration data were logarithmically processed and sophisticatedly interpolated. Moreover, the colour scale is not fixed in every colour bar of figure 8 to make comparisons among the four sub-figures. In figure 8, a darker colour indicates higher pollutant concentration. It can be concluded from the colour bar that black and red are darker than yellow and white, revealing that the concentration of the former is greater than that of the latter. Thus, it is concluded that the northwest part and the southeast part of the study area were seriously affected by the gaseous pollutant SO_2_ due to the influence of prevailing monsoons in Shanghai. By contrast, the northeast part of the study area is less polluted.

After obtaining the simulated dispersion data, concentration data at the monitoring spots of gas sensor modules extracted from the dataset serve as hard data in the SEKS-GUI software library. The total amount of the simulated monitoring data used in SEKS-GUI is 6480 entries (i.e. 60(sites) × 12(month) × 9(year)).

### 4.2. Results of BME analysis

**Figure 9.**
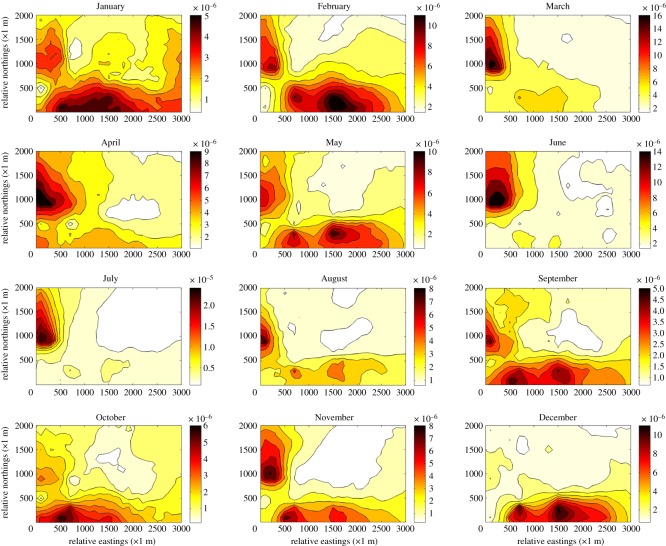
Monthly predicted mean concentration distribution of SO_2_ in 2016 by importing simulated monitoring data of gas sensor modules during the past 9 years into SEKS-GUI. (From left to right, top to bottom, the sub-figure denotes January to December in turn, the concentration is measured in μg m^−3^.)

After the first 9-year simulated dataset of monthly averaged measurements of gas sensor modules as well as geospatial information of prediction grids are loaded as inputs, the detrending stage, transformation stage and covariance analysis stage were subsequently carried out. After that, BME prediction of concentration distribution in the whole study area in the 10th year was conducted and visualization of the results is shown in figure 9. The figure illustrates the mean concentration distribution trend in every month of the last year—2016. It is worth noting that the concentration data used in this figure is the predicted raw data without interpolation and logarithmic processing. As can be seen from the figure, a darker colour indicates higher concentration of a gaseous pollutant. Obviously, it can be summarized that the northwest part of the study area was severely affected during the summer season because of the prevailing southeast monsoon, while the southeast part of the study area was severely influenced during the winter season due to the prevailing northwest monsoon. Moreover, the concrete concentration data and geospatial data of this figure are exported to the following research of designing a valid AQMN.

### 4.3. Results of multi-objective optimization model

#### 4.3.1. Results of an optimal AQMN

**Figure 10.**
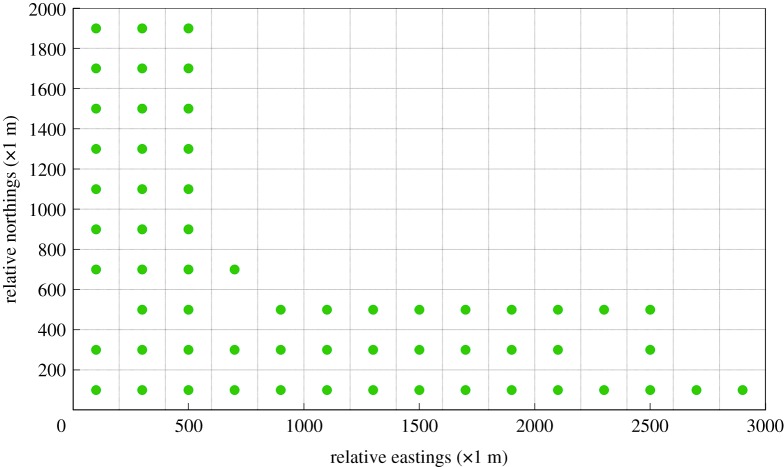
Optimal design of an AQMN on account of maximum concentration detection capability. (Green points in this figure denote gas sensor modules.)

With respect to the first objective-maximum concentration detection capability, the essence of the corresponding mixed integer linear program (MILP) is to detect the most frequent polluted grids wherein a high concentration value remarkably exceeding the standard value or the average value often occurs. After importing the dataset acquired from the BME prediction results to the corresponding MILP, the optimal design of an AQMN on account of maximum concentration detection capability is exhibited in figure 10. Compared to the initial layout of gas sensor modules, the monitoring spots which are initially positioned at the northeast part are removed in this figure. Moreover, most of the gas sensor modules are located in the northwest and southeast direction of the release spots of SO_2,_ while several gas sensor modules are positioned in the same grids or near the grids wherein the release spots of SO_2_ are located. In the Shanghai chemical industrial park, the prevailing monsoons are the summer monsoon in the southeast direction and the winter monsoon in the northwest direction. The result confirms that most concentration distribution of a specific gaseous pollutant is distributed in the downward direction of the wind. Furthermore, the layout of this optimal AQMN may result from the two release spots close to each other. A great variation in layout of this optimal AQMN would occur if the two release spots are located apart from each other.

**Figure 11.**
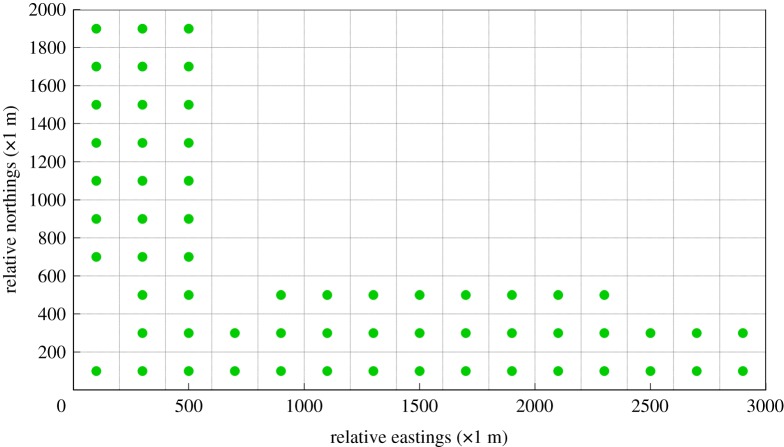
Optimal design of an AQMN on account of maximum dosage detection capability. (Green points in this figure denote gas sensor modules.)

On account of the second objective-maximum DDC, the essence of the corresponding MILP is to detect the grids wherein the accumulative dosage level is greater than the threshold of the standard value or mean value. After importing the dataset acquired from the BME prediction results to the corresponding MILP, the optimal design of an AQMN considering the target of maximum DDC is shown in figure 11. Compared to the optimal AQMN of CDC, the structure of the optimal AQMN detecting maximum dosage has barely changed. The most grids wherein long-term exposure occurs are also located in the northwest and southeast part of the chemical cluster. Slight variations in layout of the optimal AQMN appearing close to the release sources may result from the altitude intercept between the release spots and the monitoring spots. It leads to a minor possibility of high accumulative dosage values occurring at the grids which are close to the release spots.

Further, the results which reveal slight differences between the optimal AQMN of CDC and the optimal AQMN of DDC indicate that it is not necessary to combine the two objectives into a multi-objective model in this paper. However, this practice is only specific in this illustrative case study; further analysis is required to find a compromise solution between the two objectives. For example, a utility function can be derived, and then the compromise solution can be obtained by incorporating the trade-off curve. In addition, the fuzzy analytic hierarchy process method (F-AHP) can also assist the decision-making process of designing an AQMN [45]. However, such a decision analysis process is beyond the scope of this work.

Moreover, there must be some concerns about the extreme circumstances that the dispersion of gaseous pollutants may spread to the northeast part of the study area without being detected under these two optimal AQMN. This is the case, but there are also three high-accuracy air quality monitoring stations fixed in this direction. Therefore, the integrated AQMN constituted of gas sensor modules and high-accuracy fixed monitoring stations is valid and available.

#### 4.3.2. Impact of initial layout of monitoring network on predicted accuracy

**Figure 12.**
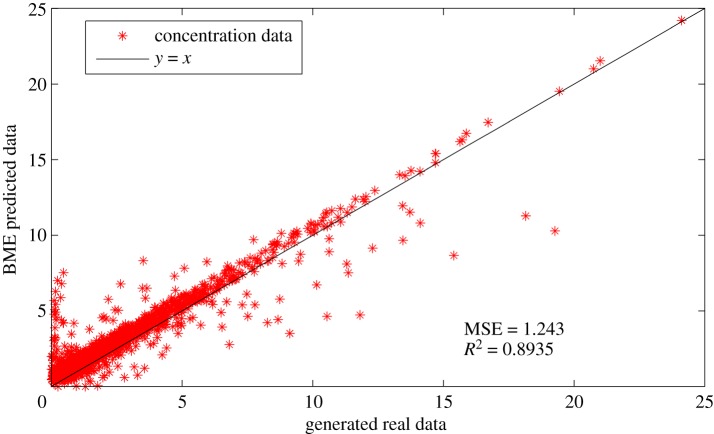
Comparison of BME-predicted data and generated simulation data when the initial layout of gas sensor modules is designed based on wind trend analysis. (The concentration unit in this figure is μg m^−3^.)

The influence of initial layout of the AQMN on prediction accuracy of BME-predicted monitoring data is tested in this subsection. As shown in figure 12, the comparison between the generated simulation data and the BME-predicted data is clearly demonstrated. Obviously, the BME-predicted data closely match the generated simulation data, the mean square error (MSE) as well as the linear regression coefficient (*R*^2^), which are 1.243 and 0.8935, respectively. The result confirms that the BME analysis can predict the measurements of unmeasured spots accurately with the information of measured spots and physical knowledge. Meanwhile, the result also indicates that the initial layout of the AQMN is reasonable for further design.

**Figure 13.**
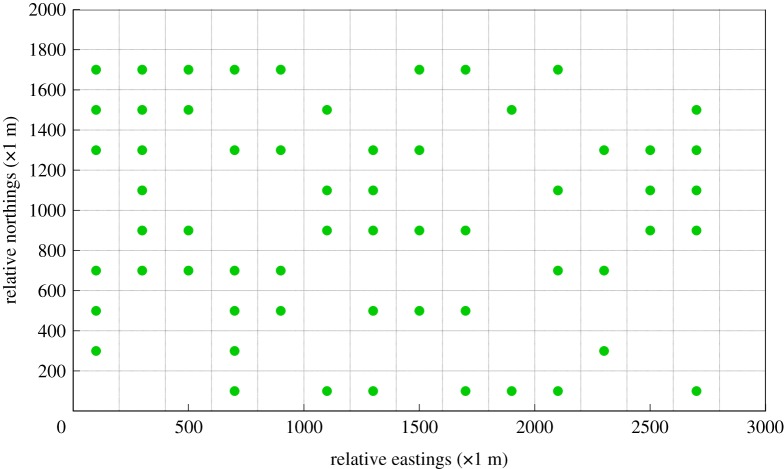
Initial random layout of gas sensor modules.

**Figure 14.**
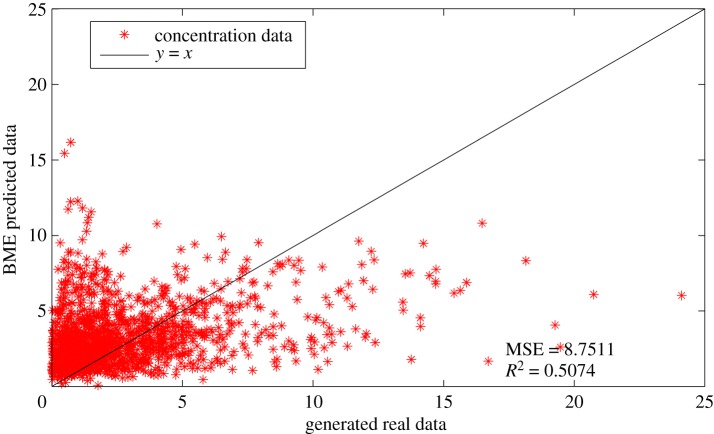
Comparison of BME-predicted data and generated simulation data when the initial layout of gas sensor modules is randomly designed. (The concentration unit in this figure is μg m^−3^.)

**Figure 15.**
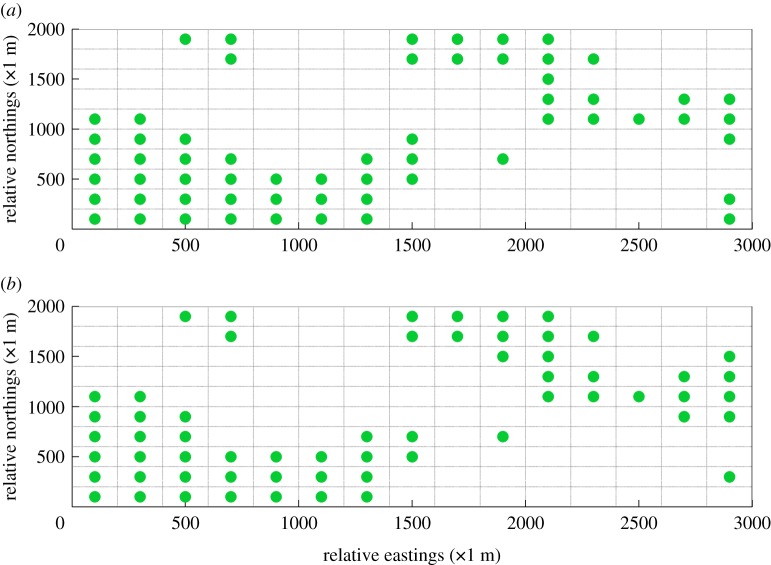
Optimal design of an AQMN when the initial layout of gas sensor modules is randomly designed. Result (*a*) is acquired based on the target of maximum concentration detection capability, while result (*b*) is acquired based on the target of maximum dosage detection capability.

If the wind field analysis is not implemented, less information is acquired about the most frequent grids in which high concentrations of gaseous pollutants would often occur. As can be seen in figure 13, the initial layout of gas sensor modules is generated by Matlab randomly. Subsequently, the first 9-year measurements of gas sensor modules at the corresponding locations were imported into the SEKS-GUI library to generate BME prediction of monitoring data at unmeasured spots in the 10th year. Moreover, the corresponding BME prediction data is also used to conduct linear regression with the generated simulation data, the comparison between which is shown in figure 14. It can be concluded that the BME prediction data is not well matched with the generated simulation data, the MSE as well as linear regression coefficient (*R*^2^) of which are 8.7511 and 0.5074, respectively. By contrast, the figures of MSE and *R*^2^ when initial layout of gas sensor modules is designed based on wind trend analysis outperform those under this randomly distributed condition. In the meantime, the corresponding optimal AQMN based on maximum concentration detection capability and maximum DDC is shown in figure 15. Comparing with the optimal layout in figures 10 and 11, the optimal layout of optimal AQMN under this random condition witnesses a contrary trend, wherein most of gas sensor modules are located in the northeast and southwest part of study area, leading to inaccurate collection of measurements.

#### 4.3.3. Impact of facility numbers of monitoring network on predicted accuracy

**Figure 16.**
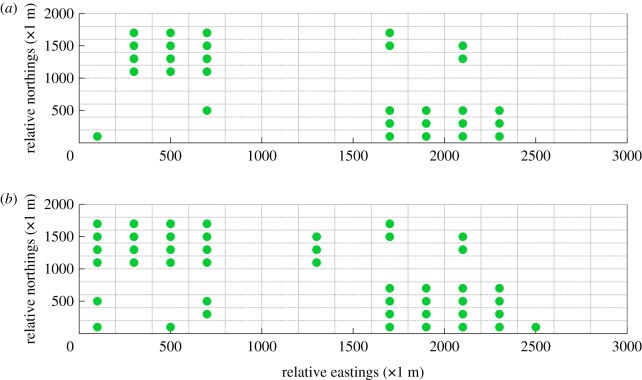
Initial layout of bounded budget scenarios. (*a*) The initial layout of the 30-facility-number scenario and (*b*) the initial layout of the 45-facility-number scenario.

**Figure 17.**
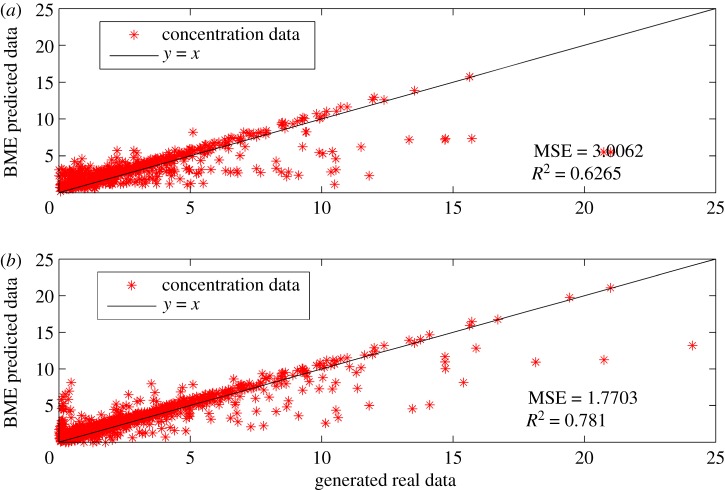
Comparison of BME-predicted data and generated simulation data under bounded budget scenarios. (*a*) The result of the 30-facility-number scenario and (*b*) the result of the 45-facility-number scenario.

In this subsection, the impact of facility numbers of monitoring network on prediction accuracy of BME-predicted monitoring data is investigated. If the environmental protection authority does not have a sufficient budget on designing such a monitoring network consisting of 60 gas sensor modules, a design plan of fewer facility numbers will be sensible. Based on results of wind trend analysis, a 30-facility-number scenario and a 45-facility-number scenario shown in figure 16 are set up. Subsequently, the first 9-year measurements of gas sensor modules at the corresponding locations (30 locations and 45 locations, respectively) were imported into the SEKS-GUI library to generate BME prediction of monitoring data at unmeasured spots in the 10th year. Moreover, the corresponding BME prediction data are also employed to conduct linear regression with the generated simulation data, the comparison between which is shown in figure 17. The figures of MSE for the 30-facility-number scenario and the 45-facility-number scenario are 3.0062 and 1.7703, respectively, while the figures of *R*^2^ in these two scenarios are 0.6265 and 0.781. Another difference in these two scenarios is that measurements of several spots are unpredictable in the 30-facility-number scenario because some areas have scarce known measured data which cannot support the prediction of reliable results at unmeasured locations. Considering the results in §4.3.2 (i.e. 1.243 for MSE and 0.8935 for *R*^2^ in 60-facility-number scenario), when the facility number of a monitoring network rises, the observable decline in MSE and the substantial increase in *R*^2^ reveal that the facility number of a monitoring network is inextricably bound up with the prediction accuracy of BME-predicted measurements, where the prediction accuracy increases as the facility number of a monitoring network rises.

### 4.4. Discussion

In this section, an illustrative case including 86 400 atmospheric dispersion scenarios of sulfur dioxide, a BME analysis experiment and the AQMN design experiments was implemented to verify the effectiveness and practicability of the proposed data-driven method for optimal design of an integrated AQMN. Through analysing the experimental results, several important findings and drawbacks could be observed and summarized as follows.

The results in §4.1, §4.2 as well as wind trend analysis reveal that the study area—Shanghai chemical cluster—is substantially influenced by the southeast monsoon and the northwest monsoon in different seasons (i.e. the wind in this area has high intensity and low direction variability). Inspired by this phenomenon, it is reasonable to select the most influenced locations where monitoring devices are deployed to increase the spatio-temporal resolution of an AQMN. The solution is pragmatic in this specific case, but probably not suitable for locations where the wind has low intensity and high direction variability.

Another finding is that the proposed data-driven method is able to assist a decision-making process for determining an appropriate AQMN, and assist the daily management work of environmental protection authorities based on results in §4.3.1. Concentration data of unmeasured spots would be accurately predicted in the BME analysis results on account of long-term measurements at monitoring sites and some physical knowledge if the initial layout of the monitoring network is well designed.

Learning from the results of §4.3.2, an astonishing finding is that the initial layout of gas sensor modules substantially affects the validity of monitoring measurements and further affects the prediction accuracy of the BME method. Moreover, it is worth noting that wind field analysis plays an important role in the initial layout of the AQMN. It ensures that most of the gas sensor modules are located in the downward direction of the prevailing wind in the study area. In addition, as time goes on, more measurements at monitoring sites can be imported to predict the variation trend in concentration distribution of gaseous pollutants, and the optimal design of an AQMN would change correspondingly, which can reflect the timely spatial and temporal characteristics of gaseous pollutants in the chemical cluster. Therefore, the data-driven method not only assists the environmental protection authorities to better inspect the chemical production activities, but also avoid the leakage of hazardous substances effectively.

Learning from the results of §4.3.3, the last finding is that the facility number of a monitoring network remarkably influences the prediction accuracy of BME predictions. It is concluded that the prediction accuracy improves as the facility number of a monitoring network increases. As a result of that, for better prediction on unmeasured spots and overall inspection on a chemical cluster, it is better to employ more inspection resources if the financial budget of the environmental protection authority is permitted.

Yet, there are some limitations in our results. First, the measurements of gas sensor modules in this study were generated from simulated scenarios. Thus, when incorporating various real-world uncertainties and constraints, the prediction accuracy of the BME analysis may change slightly. Moreover, the optimal design of an AQMN would be influenced correspondingly. Second, only one particular gaseous pollutant (i.e. sulfur dioxide) is considered in this study to design an optimal AQMN. Actually, the integrated impacts of multiple gaseous pollutants on designing an optimal AQMN should be considered. In that case, the optimal AQMN is able to collect valid measurements of a wide range of gaseous pollutants. Furthermore, it is verified that BME analysis outperforms interpolation methods mainly due to the import of soft data. Soft datasets are measurements associated with some known uncertainties, namely, faulty calibration, and long-term and short-term drifts that these monitoring devices suffer from. However, the reliability of the uncertain gas sensor module measurements is not discussed or testified in this research because of the utilization of simulated measurements (i.e. hard data refer to measurements without deviations).

## 5. Conclusion

The aim of our research is to facilitate the decision-making process of designing an integrated AQMN to inspect the chemical production processes and to assess the effectiveness of deployed pollution-controlling strategies. Previous works lack in appropriate approaches to design an integrated AQMN for a chemical industrial park. In addressing this situation, the proposed data-driven method moves forward the state of the art of this domain with the following originalities: (i) the first originality is the proposed data-driven method used, which aims to optimize the AQMN of gas sensor modules in a chemical cluster with acceptable accuracy; (ii) the second originality is to incorporate BME analysis with a multi-objective optimization method, forming a two-stage processing procedure. As the lack of long-term historical monitoring data is a major problem, our developed atmospheric dispersion simulation system was employed to generate simulated historical data based on the results of source estimation and wind analysis. Then, an illustrative case is implemented to illustrate the feasibility of the proposed approach.

Results show that the BME prediction of concentration distribution of a gaseous pollutant not only reveals the spatio-temporal distribution regularity of gaseous pollutants, but also provides essential data for designing an optimal AQMN. It is worth noting that when the initial layout of gas sensor modules is well designed, the BME-predicted data can well match the real collected monitoring data. Moreover, it is noteworthy that the prediction accuracy of BME methods has a tight connection with the facility number of a monitoring network. Increasing the facility number can greatly improve the prediction ability of the BME analysis method. Therefore, this work has been proved to have the ability to facilitate a decision-making process for determining an appropriate AQMN and assist the daily inspection work of environmental protection authorities.

Future research can lead in several directions; for instance, one can consider the impacts of multiple gaseous pollutants on designing the AQMN, or another direction for further study is the actual measurements at monitoring sites of gas sensor modules instead of simulated data.

## Data accessibility

Data and codes are attached in the supporting material files. The SEKS-GUI software library is available on the website: http://140.112.63.249/SEKSGUI/SEKSHome.html; the KD-ADSS software system is available on the homepage: http://testbot.cn/en/?page_id=7.
